# Robotic-Assisted Hepatectomy After Pancreaticoduodenectomy: A Three-Case Series

**DOI:** 10.7759/cureus.94527

**Published:** 2025-10-14

**Authors:** Akishige Kanazawa, Ayu Kosaka, Jyunya Oguma, Hiroyuki Koga, Yuichi Nagakawa

**Affiliations:** 1 Department of Gastrointestinal and Pediatric Surgery, Tokyo Medical University, Tokyo, JPN

**Keywords:** metastatic liver tumor, pancreaticoduodenectomy, previous abdominal surgeries, robotic-assisted hepatectomy, robotic liver resection

## Abstract

With the expanding use of minimally invasive hepatectomy (MIH), the proportion of patients who undergo MIH after upper abdominal surgery has been increasing. However, there are few reports showing patients who undergo MIH, particularly robot-assisted hepatectomy (RAH), after pancreatoduodenectomy (PD). Herein, we present three cases involving RAH for hepatic metastatic tumors following PD. Case 1 involves a 49-year-old male patient with metachronous liver metastasis after open PD with portal vein resection and right hemicolectomy for advanced-stage pancreatic head cancer - a solitary small liver metastasis located in the superficial part of segment 5 of the liver. RAH was performed after neoadjuvant chemotherapy. The postoperative course was uneventful, and he is currently alive without recurrence one year and six months after hepatectomy. Case 2 involves a 73-year-old female patient with metachronous liver metastasis after open PD for ampullary cancer - a solitary small liver metastasis located in the superficial part of segment 8 of the liver. RAH was performed. Despite the development of postoperative surgical site infection, she is currently alive without recurrence nine months after hepatectomy. Case 3 involves a 65-year-old female patient with metachronous liver and lung metastasis after open PD with portal vein resection for pancreatic head cancer. RAH was performed for the solitary small liver metastasis located in the superficial part of segment 5 of the liver. She was discharged on postoperative day eight, and she underwent video-assisted thoracic surgery for lung metastasis in the right middle lobe after one month. She is currently alive without recurrence six months after hepatectomy. The median surgical duration and blood loss volume were 198 (141-273) minutes and 52 mL, respectively. There were no cases of conversion. None of the patients developed severe postoperative complications, and the patients had favorable short-term outcomes. RAH after PD can be performed safely by utilizing robotic technology in carefully selected patients.

## Introduction

Hepatectomy is occasionally performed on patients with a history of upper abdominal surgery. Previous abdominal surgeries can cause abdominal adhesions and are associated with technical difficulties [1,2]. In particular, liver resection after pancreaticoduodenectomy (PD) is challenging due to extensive adhesions and the difficulty of inflow occlusion. Minimally invasive hepatectomy (MIH) is a widely used approach for benign and malignant hepatic lesions [3]. Previous studies have reported the usefulness of MIH for repeated hepatectomy utilizing pneumoperitoneal pressure and the magnified view of the laparoscope [4]. Recently, robotic surgery has become widespread and is now being performed in various fields of medicine. The use of robot-assisted hepatectomy (RAH) has been gradually reported, and RAH has shown several advantages, which include a lower volume of blood, shorter recovery time, and fewer postoperative complications related to adhesion formation compared with conventional open surgery [5-7]. At our institution, we actively perform PD for pancreatic and bile duct cancers [8], and the number of cases undergoing liver resection with robotic assistance is increasing [9]. However, evidence regarding the safety and feasibility of RAH after PD remains scarce. We aimed to describe perioperative outcomes of RAH after PD in a focused case series and to discuss selection criteria that may enable safe adoption.

## Case presentation

Surgical technique and perioperative management

We perform PD with reconstruction modified Child method and duodenal-jejunal anastomosis performed antecolic under small laparotomy [8] and routinely use anti-adhesion agents. For the RAH, all patients underwent a helical computed tomography scan of the chest, abdomen, and pelvis for preoperative staging. In most cases, this was supplemented by magnetic resonance imaging of the liver. In all cases of suspected malignancy, a pre- and postoperative discussion was held during our multi-disciplinary tumor conference, and a surgical decision was made based on the discretion of our oncologists. The decision to utilize the robotic approach was made individually between the surgical team and the patient. Informed consent about the surgery and the prospective clinical study, including disclosure about the novelty of the method and pending outcomes from larger clinical trials, was obtained from each patient. The da Vinci Xi surgical system (Intuitive, Sunnyvale, CA) was used in all surgeries. The patients were placed in the supine (French) position. Patients with lesions located in the right lateral sector were placed in the left lateral decubitus position. Trocars were inserted using the open technique, and a continuous carbon dioxide pneumoperitoneum was induced at a pressure of < 10 mmHg to prevent gas embolism. After positioning the patient in the reverse Trendelenburg position, additional trocars were placed, and standard diagnostic and staging laparoscopy were performed. In cases where trocar placement is difficult due to intra-abdominal adhesions, we perform adhesiolysis at the site laparoscopically before inserting the trocar.

After rolling in the da Vinci Xi surgical system, intraoperative ultrasonography was performed to evaluate and determine the tumor status and to assist in liver resection.

For intra-abdominal adhesion, as in the case with a history of upper abdominal surgery, meticulous dissection utilizing the robot’s forceps, including multi-joint and stabilization in addition to pneumoperitoneum pressure and magnified view, could be performed safely and securely. In particular, it could be reasonable to perform a dissection of the tangential direction between the adhered intestine with the abdominal wall. The hepatoduodenal ligament was routinely isolated and taped for the preparation of hepatic inflow occlusion (intermittent Pringle maneuver), which was used according to the surgeon’s individual decision for a maximal period of 15 minutes with five minutes of reperfusion in between. In cases after PD, hepatic inflow occlusion was thought to be difficult due to choledocho-jejunostomy or concomitant portal vein resection and reconstruction. Thus, rather than MIH, open hepatectomy may be selected for cases indicated for major hepatectomy to ensure safety in our hospital.

During hepatectomy, we routinely request a suitable central venous pressure and positive-end expiratory pressure from the anesthesiologist to control hepatic venous bleeding. The liver parenchyma was dissected with the modified clamp crush technique using Maryland bipolar electrocoagulation forceps and Fenestrated bipolar electrocoagulation forceps. The assistant surgeon was assisted mainly during hemostasis and suction by two assistant trocars. Smaller vessels were directly dissected via bipolar electrocoagulation of the da Vinci Xi surgical system. Meanwhile, those larger than the coagulating shear (particularly those > 3 mm) were cautiously ligated using titanium or Hem-o-lok clips. For extremely thick vessels, such as the right hepatic vein, left hepatic vein, and middle hepatic vein, autosutures were used. The resected, undivided specimen was placed in a plastic bag and externalized through the slightly enlarged port site. One closed drain was inserted close to the cut surface of the liver parenchyma before closing the abdominal wound. Before the subcutaneous tissue was closed, the wound was washed with saline. An absorbable suture material was used during abdominal wound closure. A drain was removed on the second postoperative day based on the quality of the secretion.

Case presentation

The three presented cases are consecutive surgical cases performed at Tokyo Medical University from March 2024 to March 2025.

Case 1

**Figure 1:**
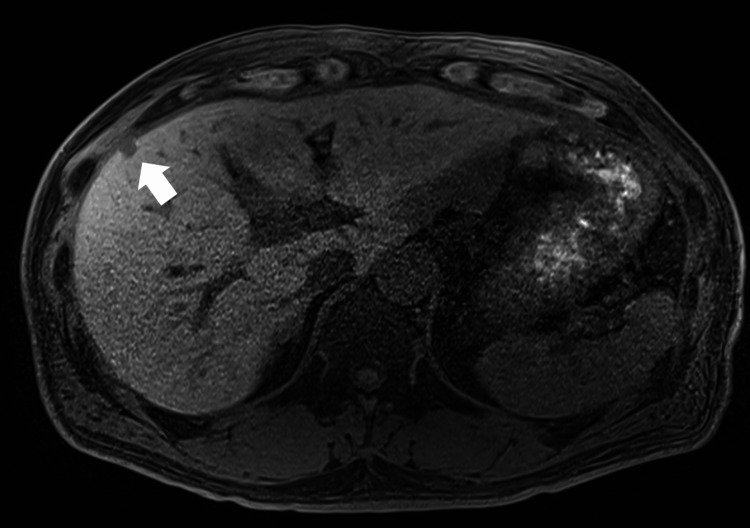
Magnetic resonance imaging T1 hepatobiliary phase of Case 1 revealed a small low-intensity mass (arrow) on the surface layer in segment 5 of the liver.

A 49-year-old male patient underwent open PD with portal vein resection and right hemicolectomy for pancreatic head cancer and subsequently received adjuvant chemotherapy. After two years, liver metastasis was identified in segment 5 of the liver (Figure 1). 

Systemic chemotherapy using gemcitabine (GEM) + nab-paclitaxel (nab-PTX) therapy (GnP therapy) was performed for 23 cycles. No new lesions were detected, and the liver metastases showed a tendency to shrink, leading to liver resection. So hepatic resection was recommended, and RAH was performed. The surgical duration was 198 minutes, and the operative blood loss volume was 1 mL. The postoperative course was uneventful, and he was discharged on postoperative day eight. Oncologic details were R0, 10 mm margin distance, poorly-moderately differentiated adenocarcinoma, confirming pancreatic origin; invasive ductal carcinoma, poorly-moderately differentiated adenocarcinoma (ly3, v3, ne2), CA19-9/CEA trends were 14.7U/mL /3.4 ng/mL to 27.4/10.0, and Response Evaluation Criteria in Solid Tumors (RECIST) was diagnosed as stable disease (SD). He is currently alive without recurrence one year and six months after hepatectomy.

Case 2

**Figure 2:**
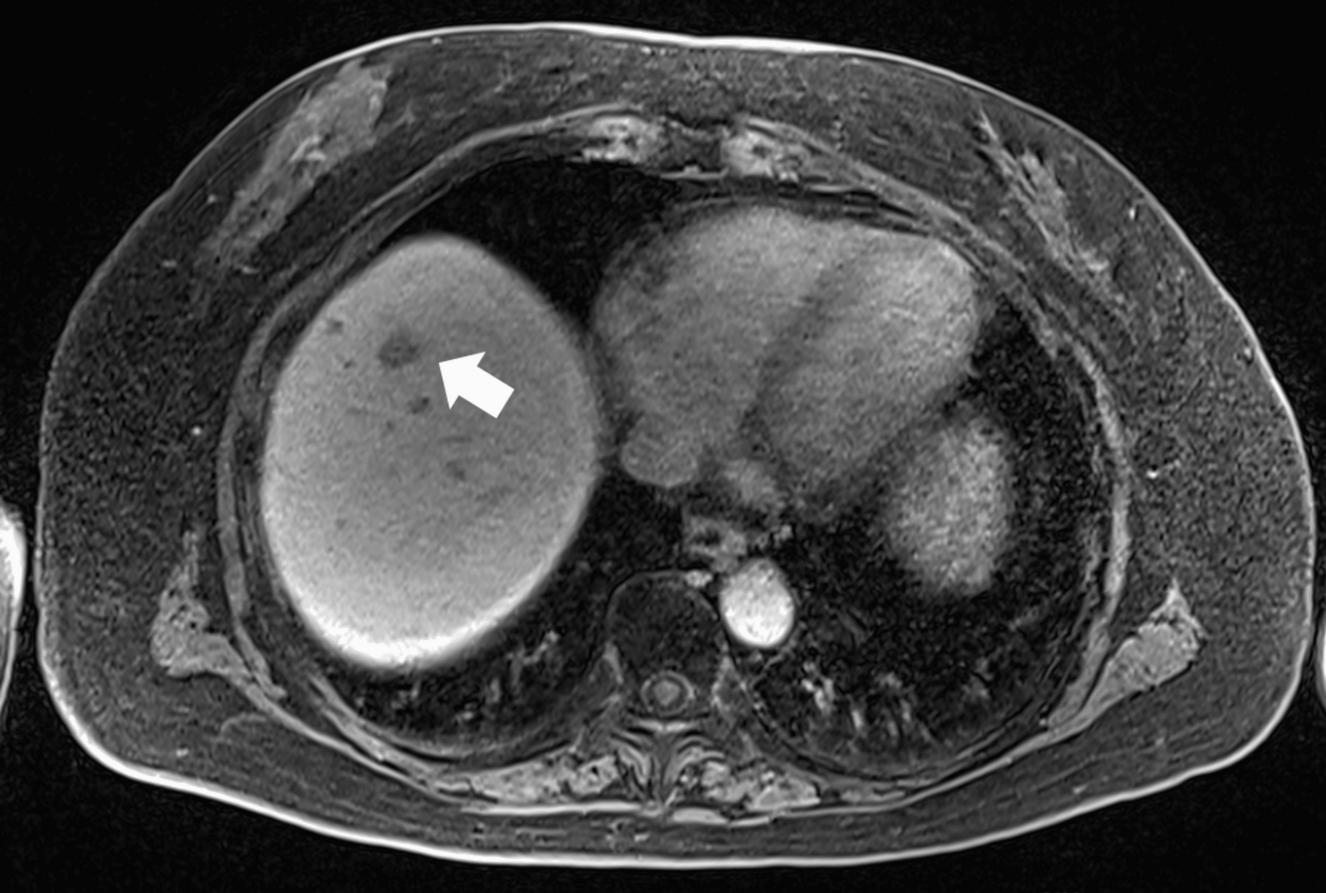
Magnetic resonance imaging T2 hepatobiliary phase of Case 2 showed a small low-intensity mass (arrow) in segment 8 of the liver.

A 73-year-old female patient underwent open PD for ampullary cancer. A 4 mm hepatic tumor lesion was detected nine months postoperatively. It was small and initially diagnosed as a postoperative liver abscess, so it was monitored. Over the next year, it slowly increased to 10 mm. Considering the slow growth rate and the possibility of a malignant tumor, hepatectomy was planned. Magnetic resonance imaging showed that the low-intensity tumor was located in segment 8 of the liver (Figure 2).

**Figure 3:**
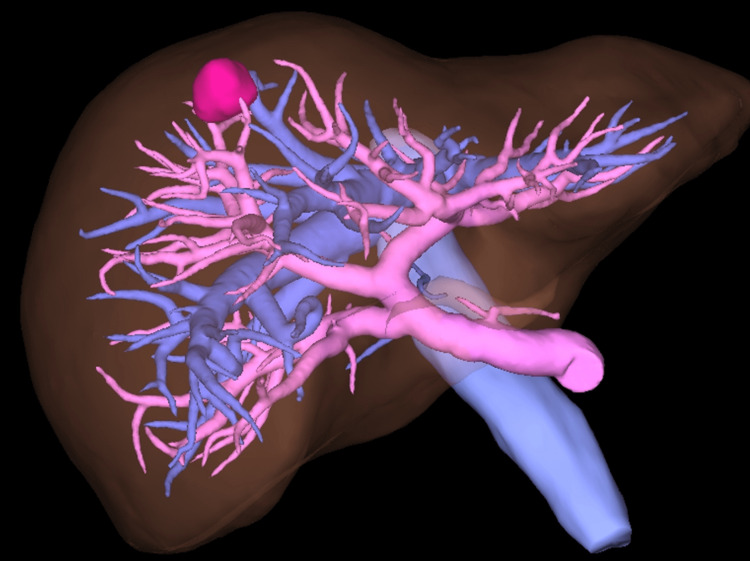
The tumor was located at the root side of the Glissonean pedicle of segment 8 G8) in the preoperative simulation image.

The tumor was located at the root side of the Glissonean pedicle of segment eight (G8) in the preoperative simulation image (Figure 3). 

RAH was then performed. During the surgery, adhesions around the first trocar site were cautiously dissected under direct vision, and the trocar was placed. Intra-abdominal adhesion was comprehensively examined. In addition to the pneumoperitoneal pressure and magnification view of the laparoscope, the robotic articulated forceps could contribute to the precise dissection of the wide adhesion, particularly the transverse colon, and abdominal wall after open PD. Intraoperative ultrasonography revealed that the tumor was located adjacent to the ventral branch of G8. As the operators had sufficient experience in laparoscopic hepatectomies without the Pringle maneuver, such as laparoscopic repeated hepatectomy [4,5], parenchymal transection could be performed safely with an assistant surgeon. After marking the resection line, parenchymal transection was performed with the modified clamp crush technique using Maryland bipolar electrocoagulation forceps and Fenestrated bipolar electrocoagulation forceps using an internal organ retractor. The target ventral branch of G8 was cautiously encircled, ligated, and dissected after double clipping with Hem-o-lock. Finally, parenchymal transection toward the right cranial side was conducted, and liver resection was completed (Video 1).

**Video 1:** Robotic-assisted hepatectomy for the metastatic liver tumor performed after pancreaticoduodenectomy for ampullary carcinoma in Case 2. This video has been approved by the patient with informed consent.

The surgical duration was 273 minutes, and the volume of blood loss was 52 mL. Despite the development of a surgical site infection (Clavien-Dindo classification II), the patient was discharged on postoperative day 18. Oncologic details were R0, 0 mm margin distance, well-moderately differentiated adenocarcinoma, confirming duodenal origin; invasive ductal carcinoma; and well-moderately differentiated adenocarcinoma. CA19-9/CEA trends were 8.5 U/mL /2.7 ng/mL to 12.2/3.7.

**Figure 4:**
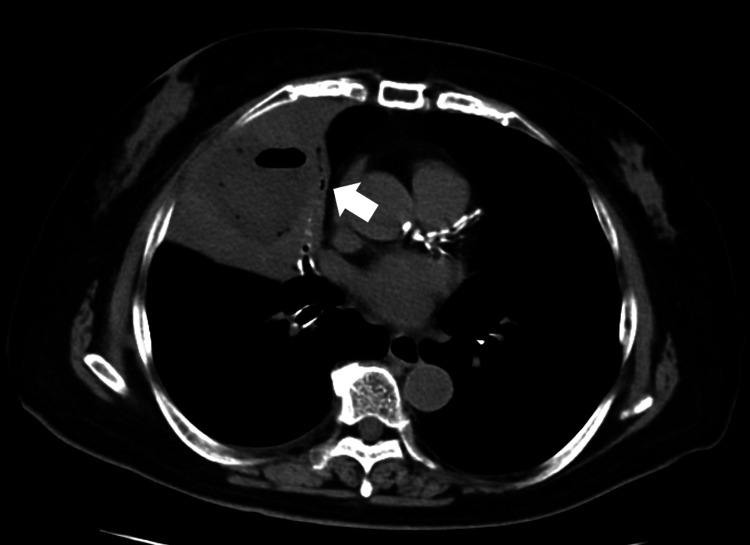
Computed tomography scan performed upon readmission of Case 2 showed the air-fluid level in the right lung proximity of the hepatic resection site (arrow).

Two months after hepatectomy, the patient was readmitted due to a lung abscess (Figure 4), which was in close proximity to the hepatic resection site.

The lesion improved with antibiotic treatment, and it was considered secondary to an abscess at the liver transection surface. The condition improved with conservative treatment. She was discharged on postoperative day 10 without complications, and she remains recurrence-free nine months after hepatectomy.

Case 3

**Figure 5:**
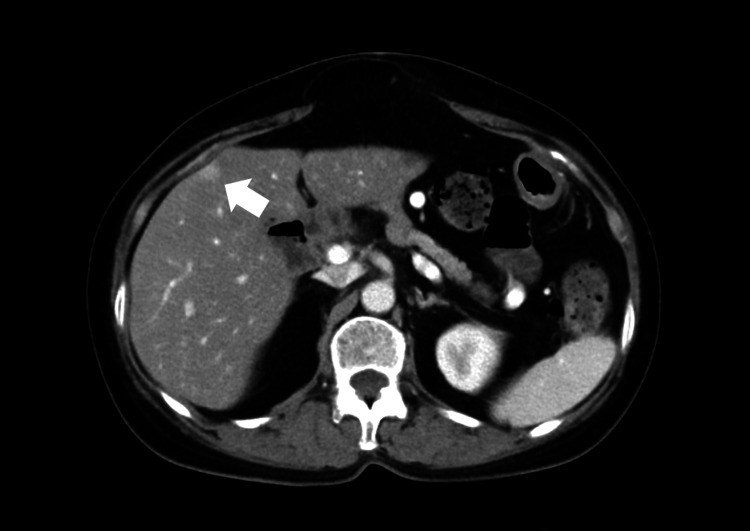
Computed tomography scan of Case 3 revealed a small early enhancement mass (arrow) on the surface layer in segment 5 of the liver.

A 65-year-old female patient presented with a metastatic liver tumor that developed after open PD with portal vein resection for pancreatic head cancer and subsequent adjuvant postoperative chemotherapy. Lung metastasis was detected 14 months after PD, and two courses of S-1 therapy were initiated. As liver metastasis was detected two months later, second-line chemotherapy with GnP therapy was introduced. Both the pulmonary and hepatic metastatic lesions showed a tendency to shrink after eight courses of chemotherapy; hepatic resection was recommended. CT showed the tumor was located in segment 5 of the liver (Figure 5). 

RAH was performed. The surgical duration was 161 minutes, and the operative blood loss volume was 197 mL. The patient was discharged on postoperative day eight without complications. Oncologic details were R0, 15 mm margin distance, poorly-moderately differentiated adenocarcinoma, confirming pancreatic origin, and well differentiated adenocarcinoma (ly2, v2, ne2). CA19-9/CEA trends were 31 U/mL /4.8 ng/mL to 32.8/4.6, and RECIST was diagnosed as SD. She underwent video-assisted thoracic surgery for lung metastasis in the right middle lobe after one month. She was discharged on postoperative day five without complications, and she is currently alive without recurrence six months after hepatectomy.

The median surgical duration and blood loss volume were 198 (141-273) minutes and 52 mL, respectively. No severe postoperative complications or recurrence have been observed, and the patients had favorable surgical outcomes (Table 1) and oncological outcomes (Table 2).

**Table 1:** Surgical outcomes of the robotic-assisted hepatectomy for metastatic liver tumors performed after pancreaticoduodenectomy. PD, pancreatoduodenectomy; SSI, surgical site infection

Case	Diagnosis	Previous surgery	Site of hepatic metastasis	Tumor size (mm)	Surgical duration (mm)	Blood loss volume (mL)	Complication	Length of hospital stay (days)
1	Pancreatic cancer	Open PD	Segment 5	13	198	1	None	8
2	Ampullary cancer	Open PD	Segment 8	20	273	52	SSI	17
3	Pancreatic cancer	Open PD	Segment 5	15	141	197	None	8

**Table 2:** Oncological outcomes of the robotic-assisted hepatectomy for metastatic liver tumors performed after pancreaticoduodenectomy. RECIST, Response Evaluation Criteria in Solid Tumors

Case	Diagnosis	R-status	Margin distance (mm)	Margin distance (mm)	Preoperative chemotherapy	Pre CA19-9 (U/mL)	Post CA19-9 (U/mL)	Pre CEA (ng/mL)	Post CEA (ng/mL)	RECIST	Prognosis
1	Pancreatic cancer	R0	10	10	Yes	14.7	27.4	3.4	10	SDF	Free survival at 16months
2	Ampullary cancer	R1	0	20	No		12.2		3.7	-	Free survival at 7months
3	Pancreatic cancer	R0	15	15	Yes	31	32.8	4.8	4.6	SD	Free survival at 4months

## Discussion

The current guidelines do not recommend liver resection as the first-line treatment for liver metastases from pancreatic or biliary tract cancer; systemic chemotherapy is generally the standard approach [10]. There is no clear evidence regarding the efficacy of resection of liver metastatic lesions after PD, and its indications are believed to be limited. It emphasized that evidence is still lacking, multidisciplinary selection criteria are necessary, and systemic therapy should be integrated.

According to previous reports, metachronous liver metastases have a significantly better prognosis compared with synchronous ones (11.4 vs. 9.1 months; p = 0.038) [11]. In addition, Tsutsumi et al. [12] reported cases of long-term survival following metastatic liver resection after pancreatic cancer surgery. Based on this finding, such procedures may contribute to improved prognosis in some cases.

Case reports and accumulating case series increasingly demonstrate that robotic surgery offers advantages, including multi-joint and stabilization in addition to 3D visualization in patients with prior upper abdominal surgery [13]. The three cases in which we performed RAH involved the following: (1) post-open PD, (2) metachronous liver metastases, and (3) small tumors measuring < 2 cm located on the liver surface.

However, for tumors on the liver surface, such as those in segments S7, S2, and S1, as well as tumors near the hepatic hilum near the choledochojejunostomy anastomosis, RAH might be applicable, with its various functional advantages might be particularly beneficial in selected cases.

These factors could have contributed to the safe performance of RAH and the achievement of favorable long-term outcomes. Previous abdominal surgery may cause abdominal adhesions. In particular, the laparoscopic approach is technically challenging [1,2]. However, Feldbrügge et al. [14] showed that a history of abdominal surgery is not a risk factor for postoperative complications after RAH. They reported that there were no significant differences in terms of surgical duration, conversion rate, or presence of severe postoperative complications between patients with a history of upper abdominal surgery and those without [15]. Univariate analysis revealed that patients with a history of liver resection had a longer surgical duration. Therefore, RAH may be beneficial for cases with a history of upper abdominal surgery. However, there are no reported cases of RAH after PD, which is one of the most extensive upper abdominal surgeries. RAH was performed for small tumors. Even in cases of PD with extensive adhesions, liver resection was performed by peeling only the necessary areas of adhesions using the robot’s multi-jointed forceps, with good short-term postoperative outcomes.

After PD, hepatic inflow occlusion is considered difficult due to dissection around the hepatoduodenal ligament, choledocho-jejunostomy, and concomitant portal vein resection and reconstruction. Even in cases where the Pringle maneuver was challenging, safe liver resection was performed using pneumoperitoneum pressure, precise liver resection maneuvers, and assistant support. In this case series, the Pringle maneuver was not applied. The three cases at our hospital involved small tumors measuring < 2 cm that are located on the liver surface. Further, the use of adhesion prevention sheets after open PD surgery and our extensive experience with MIH, including robot-assisted procedures, may have contributed to the favorable short-term outcomes. However, all cases in this study involved relatively small partial resections, and it remains unclear whether MIH is applicable in cases requiring extensive liver resection.

Although ablation therapies such as RFA after biliary reconstruction are known to be less invasive procedures, it has been reported to carry a high risk of postoperative liver abscess and are therefore not recommended [16].

One case required readmission due to a lung abscess that was believed to have spread from a liver resection site abscess. Fortunately, the condition improved with conservative therapy using antibiotics. An infectious complication of the liver is one of the main issues associated with hepatectomy after PD [17,18]. Enteric bacterial colonization in the bile with biliary-enteric anastomosis is indicated, resulting in abscess formation after liver resection [18]. In our case, it is assumed that the enteric bacteria from biliary-enteric anastomosis infected the cut surface of segment 8 of the liver, and it might have spread transdiaphragmatically to the lung and formed a lung abscess. Therefore, more attention should be paid to late infection after liver resection with biliary-enteric anastomosis compared with that after liver resection without biliary-enteric anastomosis, such as confirming biliary bacterial culture during the stent placement, appropriate use of the targeted antibiotics, selective drain use, early imaging for fever, and criteria for drain removal.

## Conclusions

Although MIH is challenging due to extensive adhesions and the difficulty of inflow occlusion for cases of metastatic liver tumors after PD, RAH could be performed safely by utilizing robotic technology and provide feasible short-term outcomes. Thus, it appears safe and feasible, demonstrating its favorable short-term outcomes (6-18 months) in carefully selected patients with small, superficial, late-stage metastases following PD. Next step studies would be proposed, such as propensity-matched comparison, time-to-systemic-therapy, quality-of-life, and cost/resource analysis.
